# The relationship between self-compassion and mental health: A repeated measures investigation

**DOI:** 10.1371/journal.pmen.0000548

**Published:** 2026-04-01

**Authors:** Ming Yu Claudia Wong, Janet Yuen-Ha Wong, Stanley Kam Ki Lam, Hong Wang Fung

**Affiliations:** 1 Department of Health and Physical Education, The Education University of Hong Kong, Hong Kong; 2 School of Nursing and Health Sciences, Hong Kong Metropolitan University, Hong Kong; 3 School of Nursing and Mental Health Research Centre, The Hong Kong Polytechnic University, Hong Kong; National University of Singapore, SINGAPORE

## Abstract

Self-compassion is a significant factor in mental health outcomes across various populations. Research has shown that self-compassion may improve mental health by promoting emotion regulation and resilience, as well as protecting people from depression and anxiety. Despite sufficient research on self-compassion and mental health, most current studies fail to demonstrate causality or track changes over time. Moreover, there is a significant gap in existing research regarding the use of SEM or path models to conceptualize the interplay among self-compassion components. By conceptualizing how these components interact, path models can provide deeper insights into the mechanisms through which self-compassion exerts its effects. This repeated measures study analyzed data from 232 participants who completed valid measures at two time points. The aim was to examine the relationship between self-compassion and mental well-being, as assessed by a validated self-rated measure of mental health (SRMH). Additionally, the study examined how distinct facets of self-compassion contributed uniquely to SRMH over time. Results revealed that the proposed models demonstrated a good fit to the data. This confirmed the differential predictive abilities of separate self-compassion dimensions regarding subsequent SRMH. Specifically, self-kindness emerged as the strongest predictor of positive mental health in subsequent analyses. In addition, mediating variables such as common humanity and isolation were identified as significant pathways through which self-kindness and over-identification influenced SRMH. Moreover, applying repeated measures panel analysis within a cross-lagged framework further substantiated this finding. Overall, the results supported consistent associations between facets of self-compassion and mental well-being across two measurement occasions. Based on these results, future studies should investigate interventions to cultivate self-kindness actions, such as through loving-kindness meditation or compassion-focused exercises. These methods are expected to be impactful in treating and preventing various mental health issues by embracing self-kindness and lessening isolation.

## Introduction

Self‑compassion is defined as the ability to treat oneself with kindness in times of suffering, to recognize one’s experiences as part of the shared human condition, and to maintain mindful awareness of distress without over‑identification [1,2]. The importance of this work lies in clarifying how self-compassion operates over time, since although its link to mental health is well established, the mechanisms and sequencing of its components remain unclear. The importance of this work lies in clarifying how self-compassion operates over time, since although its link to mental health is well established, the mechanisms and sequencing of its components remain unclear. This rationale underpins the present study, which seeks to explore whether specific facets of self-compassion differentially predict mental health outcomes. Self-compassion has been proposed as an essential protective factor for mental health, with recent large-scale studies indicating that self-compassion is associated with positive mental health and life satisfaction among emerging adults [3,4]. Numerous studies have established a strong connection between high levels of self-compassion and better mental health outcomes. [5] suggests that self-compassion boosts psychological well-being by improving emotional resilience and stability, serving as a key protective factor against mental health problems like depression and anxiety. Long-term research with 391 university students who had been left-behind children indicates that self-compassion may be an important protective factor in preventing depression, especially among those who tend to avoid emotional experiences [6]. Studies examining individuals with high self-compassion traits have demonstrated that these traits are positively associated with several mental health metrics, including reduced neuroticism and depression, as well as increased life satisfaction, social connections, and overall well-being [7]. Further, a study involving 31 participants who completed Compassion Focused Therapy (CFT) identified self-compassion as a valuable coping resource, effectively reducing catastrophic thinking, anxiety, and avoidance behaviors in adverse situations [8]. Besides mental health, a meta-analysis [9] reported a correlation of r = .47 between self-compassion and well-being, with stronger associations noted in cognitive and psychological dimensions than in affective well-being. The study further examined sample characteristics and self-esteem as moderators and found that a subsample of the research demonstrated a causal relationship between self-compassion and improvements in well-being. Most current studies fail to demonstrate temporal change or directional associations over time. While causality cannot be fully established with two timepoints, longitudinal modelling provides stronger evidence of temporal ordering than cross-sectional designs.

Self-compassion is often described in scholarly works as the unbiased embrace of one’s own distress, coupled with showing kindness to oneself [10,5]. Influenced by Buddhist philosophy, Neff [1] described self-compassion as consisting of three parts: self-kindness, understanding during hardships rather than self-criticism; common humanity, acknowledging one’s experiences as universal rather than isolating; and mindfulness, maintaining balanced awareness of adverse emotions to mitigate the impacts of self-judgment, isolation, and rumination. [2] explicitly note that “although these three components of self-compassion are conceptually distinct, they overlap and tend to engender one another,” operating not as isolated domains but rather as elements of a dynamic, synergistic system. Negative components of self‑compassion, such as self‑judgment and isolation, have been linked to poorer psychological outcomes [11,12]. These constructions represent maladaptive processes that may undermine mental health, making their inclusion in the present study essential. Recent empirical and theoretical work has begun to elucidate the process-like nature of these relationships. For instance, [13] highlight that mindfulness—particularly the balanced awareness of negative thoughts and feelings—is not only an essential component of self-compassion but often functions as a precursor to the other two elements, enabling individuals to approach suffering with kindness and a sense of shared humanity. Nevertheless, while several studies have examined the collective effect of self-kindness, common humanity, and mindfulness on self-compassion, the relative contributions of these components and the optimal sequencing of their application remain unclear. While recent exploratory work has begun to investigate potential sequencing among components, Neff’s original self-compassion theory emphasizes three synergistic components—self-kindness, common humanity, and mindfulness—that interact dynamically rather than sequentially. The present study incorporates this theory by testing both synergy-based and exploratory sequential models, thereby examining whether empirical data support the original conceptualization or suggesting alternative pathways. and there is no established evidence supporting a staged progression, where optimal mental well-being may progress from mindful awareness to self-reflection and judgment (guided by kindness), and ultimately to feelings of common humanity instead of isolation [14]. Although Neff’s foundational work defines self-compassion as comprising three synergistic facets, the processual ordering of these facets remains theoretically unresolved. The current study adopts an exploratory approach, systematically testing alternative sequences to determine which, if any, yield optimal mental health outcomes.

To address this gap, the present study aims to systematically examine which specific component of self-compassion—self-kindness, common humanity, or mindfulness—serves as the strongest predictor of mental health outcomes. Furthermore, this research seeks to explore and clarify how the potential process stages of these three components unfold, using longitudinal path analysis to capture the dynamic interactions and causal ordering over time. Understanding the stage processing pathway between self‑compassion components is important because interventions may be more effective if they target the most influential or foundational component first. For example, cultivating self‑kindness may facilitate later development of common humanity, thereby strengthening resilience and reducing isolation. Clarifying these pathways provides theoretical guidance for sequencing intervention strategies and practical implications for clinical programs. The present study’s approach to modeling sequential relationships and testing hypothetical orders remains strictly exploratory and hypothesis-generating. Any findings regarding optimal sequencing or pathway dynamics should be interpreted within this context, rather than as theory-driven or prescriptive conclusions in line with Neff’s original synergy-based model. Clarifying these differential effects will provide theoretical and practical guidance for sharpening self-compassion interventions targeting mental health. While recent longitudinal investigations [15,16,p. 202,^17^], have begun to address longstanding limitations of cross-sectional research, most extant studies remain unable to demonstrate true causality or temporal change. The few available longitudinal studies typically suffer from constraints such as small sample sizes or short assessment periods. Thus, robust, longitudinal path-analytic approaches, including cross-lagged modeling—are crucial for untangling the time-ordered relations among the psychological constructs involved.

By utilizing these longitudinal and structural methods, this study seeks not only to illuminate the mechanisms by which self-compassion influences well-being over time but also to inform the design of more targeted mental health interventions. In addition, a deeper understanding of these component interactions and their process dynamics may inspire future research exploring cultural differences, integrating self-compassion with other psychological constructs, and advancing clinical trials that evaluate self-compassion as a therapeutic mechanism.

## Purpose of the study

- To investigate the relationship between self-compassion and mental well-being (as measured by a well-validated tool of self-rated mental health [SRMH], as well as how each of the components of self-compassion is related to SRMH.
- To explore and investigate the stage processing pathway between components of self-compassion
- To investigate the consistency and interaction between the two points for the robustness of findings using path analysis with cross-lagged effects.

## Path analysis model hypothesis

**Hypothesis 1:** Self-compassion predicts self-rated mental health in a hierarchical model and demonstrates a good-fit model.

**Hypothesis 2:** Common humanity and self-kindness mediate the relationship between mindfulness and self-rated mental health, as per the good-fit model (Mindfulness → Common Humanity → Self-kindness → Mental Health).

**Hypothesis 2a:** Common humanity and self-kindness mediate the relationship between mindfulness and self-rated mental health, as per the good-fit model (Mindfulness ◊ Self-kindness ◊ Common Humanity ◊ Mental Health).

**Hypothesis 3:** Self-judgement and Isolation mediate the relationship between over-identification and self-rated mental health with the good-fit model (Over-identification ◊ Self-judgement ◊ Isolation ◊ (negative) Mental Health).

All hypothesized models are performed in both path analysis for the single time point and repeated measures panel analysis for the two-time point. We acknowledge that the study is exploratory, yet hypotheses were generated to provide structure. Mindfulness was positioned at the start of pathways because prior literature suggests it enables balanced awareness of distress, which may facilitate subsequent self-kindness and common humanity [13].

## Methodology

This study analyzed data from a longitudinal survey, which obtained ethical approval from the institutional review board at the Chinese university of Hong Kong. From 30/11/2022 to 30/05/2023, potential participants were recruited using advertising through social media platforms (i.e., Facebook and Instagram). The inclusion criteria, which were checked at the beginning of the online survey, included the following: be between 18–64 years old, provide online written informed consent, voluntarily agree to participate, be a current Hong Kong resident, be able to read and write Chinese, and have access to the web-based surveys. The inclusion criteria were intentionally broad to capture a diverse community sample of Hong Kong adults. Given the modest sample size, broad criteria maximized recruitment feasibility while ensuring sufficient statistical power for path analysis. Participants with learning or reading disorders, dementia, or cognitive impairments were excluded from the study. Individuals with severe cognitive impairments or learning disorders were excluded because the study relied on self-report measures requiring intact comprehension. However, we acknowledge that excluding people with disabilities limits generalizability. This lack of disability representation is noted as a limitation.

Participants completed self-report psychological assessments at baseline on the online survey. After approximately six months, they were invited to complete a follow-up online survey again.

A total of 232 participants provided valid data at both timepoints. Their ages ranged from 18 to 64 (M = 39.65; SD = 12.75). Most of them were female (82.3%), full-time employed (56.5%) or part-time employed (19.0%), were not married (62.5%), and had a bachelor’s degree (59.1%). At baseline, 28.9% reported receiving psychiatric services within the past 12 months. Table 1 shows the demographic characteristics of participants.

**Table 1:** Demographic characteristics of participants.

Variables	n (%) or M ± SD
Age (years)	39.65 ± 12.75
Gender (Female)	191 (82.3%)
Employment (Full-time)	131 (56.5%)
Employment (Part-time)	44 (19.0%)
Marital Status (Single)	145 (62.5%)
Education (Bachelor’s)	137 (59.1%)
Psychiatric services (past 12 months)	67 (28.9%)

It presents demographic characteristics of the sample, including age, gender, marital status, education, and psychiatric service use.

The methodology and part of the data unrelated to the hypotheses have been reported elsewhere [18]. A common rule of thumb suggests a minimum of 200 participants for stable parameter estimates in SEM [19]. Additionally, a sample size of at least 10 participants per estimated parameter is recommended [20]. Given a typical cross-lagged model with two variables across two timepoints (e.g., estimating paths, variances, and covariances), approximately 10–20 parameters may be estimated, requiring a sample size of 100–200. The current sample of 232 exceeds these thresholds, providing adequate power to detect moderate effect sizes while accounting for model complexity and ensuring reliable estimates.

### Measures

Self-compassion was measured using the 26-item Self-Compassion Scale (SCS), a self-report measure with excellent reliability. The scale comprises six factors: self-kindness, common humanity, mindfulness, self-judgment, isolation, and overidentification [21]. [22] validated the Chinese version of the SCS, confirming its robust internal consistency (α = .84), construct validity, and the retention of the six-factor structure.

Mental health was assessed using the single-item measure of self-rated mental health (SRMH). This measure, which asked, “How would you rate your overall mental health?” (1 = poor, 2 = fair, 3 = good, 4 = very good, 5 = excellent), has been commonly used in epidemiological studies to assess global mental health [Ahmad et al., 2014]. The Chinese version of the measure was reported to have good test-retest reliability (ICC = .75), a strong positive correlation with self-esteem, as well as a strong negative correlation with mental health symptoms (e.g., depressive symptoms and post-traumatic stress) across samples [23]. Therefore, this measure was used as an indicator of mental health in the present study.

### Author agreement and statements

**Ethics approval:** The paper analyzed data from a survey project, which obtained ethical approval from the institutional review board at the Chinese University of Hong Kong. All participants provided online written informed consent before they completed the survey. Survey and Behavioral Research Ethics Reference No. SBRE‐22‐0051 on Nov 08, 2022, at The Chinese University of Hong Kong.

The dataset generated and analyzed during the current study is available upon reasonable request from the Survey and Behavioural Research Ethics Committee (Survey and Behavioral Research Ethics Reference No. SBRE‐22‐0051 on Nov 08, 2022) at The Chinese University of Hong Kong (fssc02@cuhk.edu.hk) as the durable point of contact for data requests. This ensures long-term accessibility and compliance with PLOS policy. Data is not publicly available due to ethical restrictions, but requests will be reviewed and approved by the committee to ensure long-term accessibility.

### Data analysis

Path analysis with cross-lagged effects was conducted to assess model consistency between Time 1 and Time 2. Moreover, it included the utilization of cross-lagged panel modeling (CLPM) for analyzing the direction and causality of relationships between variables measured at two time points. Confirmatory Factor Analysis was conducted for the self-compassion scale, while the reliability and validity of the single-item measure of self-rated mental health (SRMH) have been previously documented in studies by Ahani et al., [24] and Fung et al., [23]. The model fit is assessed based on several criteria. The chi-square value is required to be between 2 and 5 for a good-fitting model, but this can vary with sample size. Therefore, additional indicators will also be considered. The comparative fit index (CFI) and non-normed fit index (NNFI) should be 0.90 or greater, demonstrating a well-fitting construct validity model. The standardized root mean square residual (SRMR) should also be 0.05 or less. The root mean square error of approximation (RMSEA) value should be 0.08 or lower, with the 90% confidence interval included. Meeting these thresholds for the CFI, NNFI, SRMR and RMSEA would indicate an acceptable model fit [25,26]. Missing data were minimal (<5%). Little’s MCAR test indicated data were missing completely at random (p > .05). Missing values were handled using full information maximum likelihood (FIML) estimation, consistent with SEM best practices.

## Results

### Reliability and validity of the instrument

The self-compassion measurement model exhibited satisfactory internal consistency at both time points, with Cronbach’s α and ω values exceeding 0.70 (see Table 2). Internal consistency estimates for all six subscales of the Self-Compassion Scale (SCS) were calculated in the present study. Cronbach’s α values ranged from.72 (common humanity) to.86 (self-kindness), indicating acceptable to good reliability. Furthermore, confirmatory factor analyses confirmed excellent model fit acceptable model fit, with some indices (RMSEA, SRMR) approaching borderline thresholds for both time points: χ²(308) = 4.81, CFI = 0.99, TLI = 0.99, SRMR = 0.069, and RMSEA = 0.078 (90% CI [0.067, 0.089]).

**Table 2:** Summary of the reliability of the self-compassion subscales.

	Cronbach’s alpha	Omega
**Time 1**		
**Self-Kindness (SK)**	0.87	0.87
**Self-Judgment (SJ)**	0.85	0.85
**Common Humanity (CH)**	0.80	0.80
**Isolation (IS)**	0.78	0.78
**Mindfulness (MF)**	0.84	0.84
**Over-Identification (OI)**	0.75	0.75
**Time 2**		
**Self-Kindness (SK)**	0.90	0.90
**Self-Judgment (SJ)**	0.82	0.82
**Common Humanity (CH)**	0.80	0.80
**Isolation (IS)**	0.78	0.78
**Mindfulness (MF)**	0.85	0.85
**Over-Identification (OI)**	0.74	0.75

### Self-compassion and well-being relationship consistency

**Fig 1:**
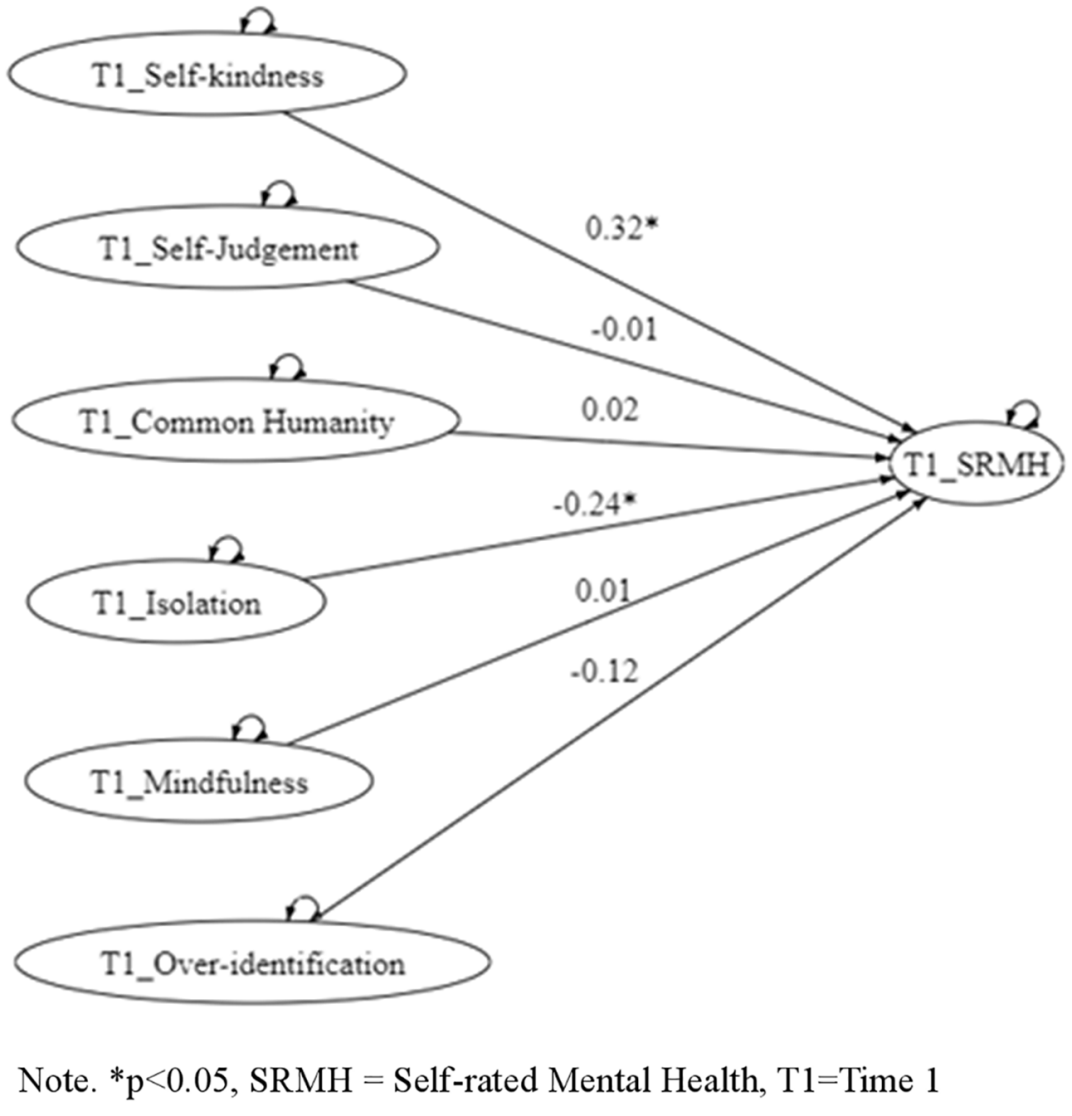
A hierarchical model indicating the association between the self-compassion components with self-rated mental health.

**Fig 2:**
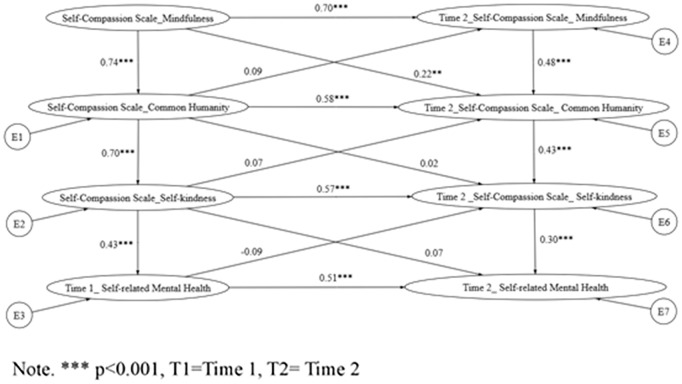
Cross-lagged model of Hypothesis 2 indicates the relationship between the positive self-compassion components and self-rated mental health.

**Fig 3:**
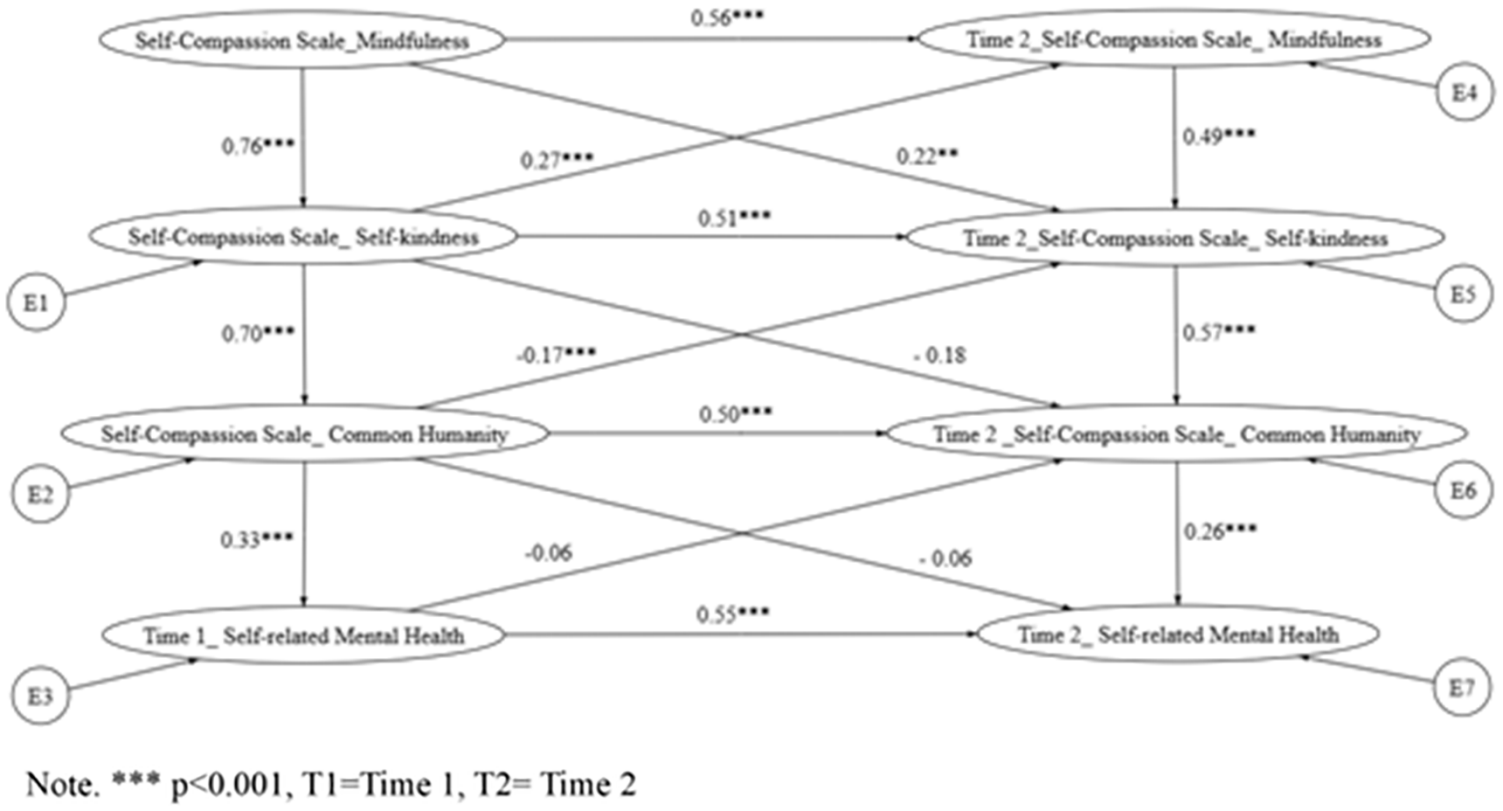
Cross-lagged model of Hypothesis 2a indicates the relationship between the positive self-compassion components and self-rated mental health.

**Fig 4:**
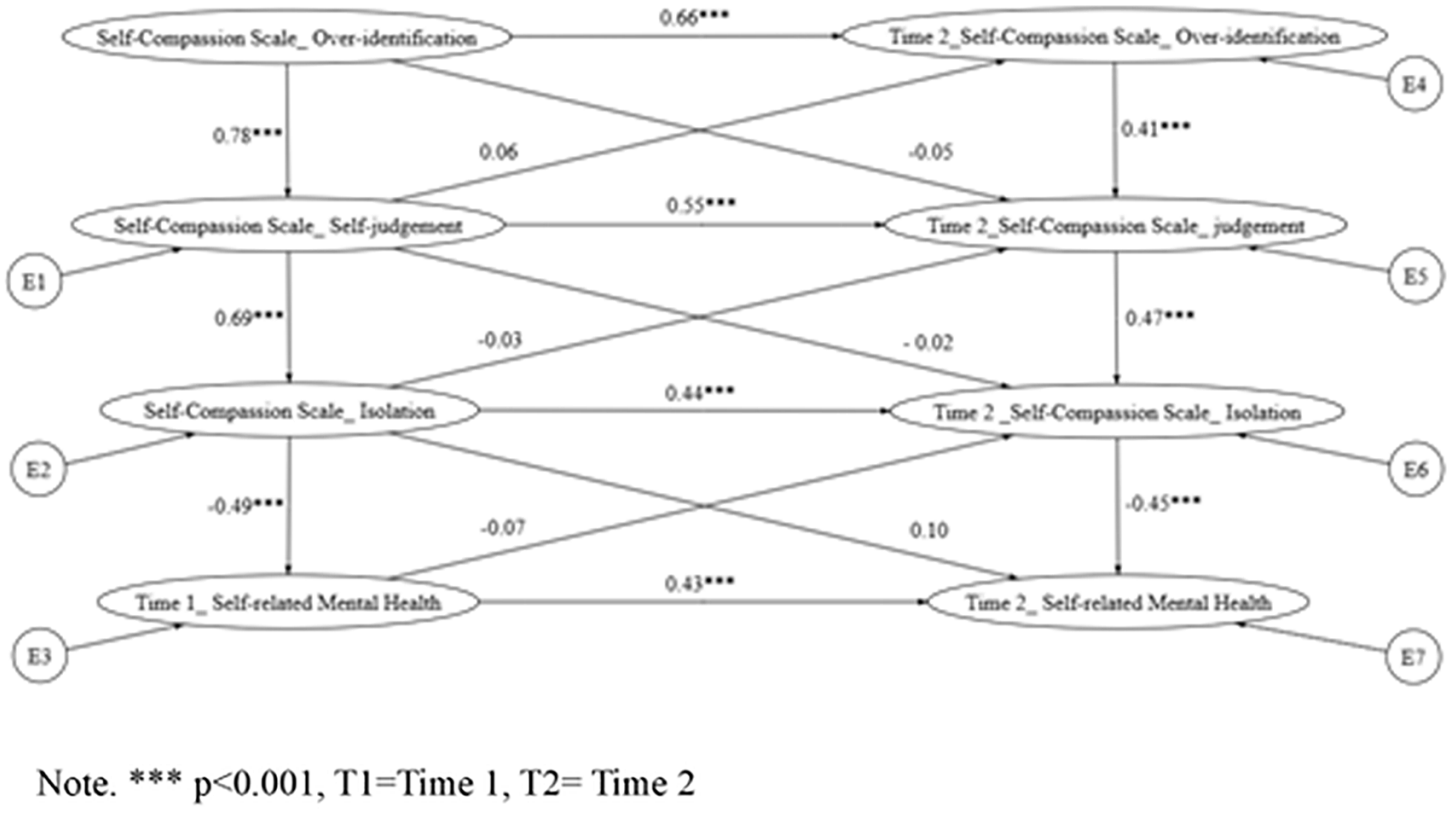
Cross-lagged model of Hypothesis 3 indicates the relationship between the negative self-compassion components and self-rated mental health.

Building on the hierarchical path models, cross-lagged panel analyses (via structural equation modeling; Fig 1) examined the directional influences among self-compassion components and SRMH across time points, as specified in Hypotheses 2 and 3. These models tested sequential processes informed by prior literature, whereby mindfulness at Time 1 fosters common humanity and/or self-kindness at Time 2, thereby enhancing SRMH. Model fit indices are summarized in Table 3. For clarity and reader accessibility, Table 3 is presented directly below alongside the explanatory text. Similarly, Figs 2–4 are embedded within the Results section to visually illustrate the tested pathways.

**Table 3:** Summary of the path analysis good fit index (hypothesis 1).

Model	Description	X^2^	df	CFI	NNFI	RMSEA	SRMR	Model AIC
**1 (Fig 1**)	Time 1 only	338/80 = 4.22	80	0.99	0.98	0.072 (CI95%0.062 -0.082)	0.070	635.09
**2**	Time 1 and Time 2 with the repeated path from Time 1 – Mental Health towards Time 2 – Mental Health	34.48/12 = 0.87	12	0.99	0.97	0.087 (CI95%0.050 -0.12)	0.022	217.67
**3**	Time 1 and Time 2 with repeated paths linked for all variables	439.64/57 = 7.71	57	0.93	0.89	0.17(CI95%0.16 -0.19)	0.12	531.59

Note. CFI - Comparative Fit Index; NNFI - Non-Normed Fit Index; RMSEA - Root

Mean Square Error of Approximation; SRMR - Standardized Root Mean Square

Residual; Model AIC – Model Akaike Information Criterion

For Hypothesis 2 (Model 4; see Fig 2), the null hypothesis was rejected (χ² p = .06). Though this model showed the poorest fit among the tested alternatives (CFI = 0.92, TLI = 0.89, RMSEA = 0.085). Nevertheless, all Time 1 variables exerted significant cross-lagged effects on their Time 2 counterparts (β ranging from 0.22 to 0.38, p < .01), supporting bidirectional influences without a specified sequential order.

In contrast, Hypothesis 2a (Model 5; see Fig 3) posited a specific sequence: self-kindness at Time 1 predicts common humanity at Time 2, which in turn predicts SRMH at Time 2. This model achieved acceptable fit (CFI = 0.94, TLI = 0.90, RMSEA = 0.072) and AIC values for Models 4–6 are now reported in Table 4, allowing direct comparison of model parsimony, indicating superior parsimony and explanatory power. The sequential paths were statistically significant: self-kindness (Time 1) → common humanity (Time 2; β = 0.31, p < .001) and common humanity (Time 1) → SRMH (Time 2; β = 0.28, p < .001), with an indirect effect of self-kindness on SRMH via common humanity (β = 0.09, p < .01; 95% CI [0.04, 0.15]). These findings provide direct empirical support for the hypothesized mindfulness-mediated pathway to improved well-being.

**Table 4:** Summary of the Good Fit Index (Hypothesis 2 and 3).

Model	Description	X^2^	df	CFI	NNFI	RMSEA	SRMR	Model AIC
**4 (Fig 2**)	MF ◊ CH ◊ SK ◊ SRMH (Cross-lagged)	130.16/ 12 = 10.85	12	0.91	0.80	0.21 (CI90% 0.175–0.239)	0.099	178.157
**5 (Fig 3**)	MF ◊ SK◊ ◊ CH ◊ SRMH (Cross-lagged)	97.49/ 12 = 8.12	12	0.94	0.90	0.18 (CI90% 0.144–0.209)	0.099	145.486
**6 (Fig 4**)	OI ◊SJ ◊ IS◊ SRMH (Cross-lagged)	109.003/ 12 = 9.08	12	0.92	0.82	0.19 (CI90% 0.15–0.22)	0.109	157.003

Note. CFI - Comparative Fit Index; NNFI - Non-Normed Fit Index; RMSEA -

Root Mean Square Error of Approximation; SRMR - Standardized Root Mean

Square Residual; Model AIC – Model Akaike Information Criterion

For Hypothesis 3 (Model 6; see Fig 4), which examined adverse effects of negative self-compassion components (isolation and over-identification), the null hypothesis was not rejected (χ² p = .08), with marginally acceptable fit (CFI = 0.93, TLI = 0.91, RMSEA = 0.074). Significant negative cross-lagged paths emerged: isolation (Time 1) → SRMH (Time 2; β = -0.26, p < .001) and over-identification (Time 1) → SRMH (Time 2; β = -0.24, p < .001). An indirect effect via isolation further evidenced its detrimental role (β = -0.08, p < .05; 95% CI [-0.14, -0.02]), aligning with theoretical expectations of reduced well-being through heightened self-criticism.

In summary, Model 5 provided the strongest evidence for positive sequential processes, while Models 4 and 6 confirmed bidirectional and negative directional effects, respectively, with all paths supported by statistically significant coefficients and fit indices.

## Discussion

The present study examined the hierarchical model’s capacity to discern associations between self-compassion and self-rated mental health (SRMH), especially among Hong Kong adults. Results indicated the model exhibited good fit, thereby upholding the differential contribution levels from the discrete facets of self-compassion towards SRMH. Notably, both self-rated mental health and the self-compassion components demonstrated considerable temporal stability, supporting the reliability of these constructs and their suitability for modeling directional relationships across time. The application of path analysis with cross-lagged effects within a longitudinal framework (referring to Figs 2–4) further substantiated this finding.

Within the hierarchical model, self-kindness was the strongest predictor of later mental health, thereby adding to the growing body of research by providing longitudinal evidence that self-kindness is a particularly influential part of self-compassion in predicting mental health outcomes. Supporting this outcome, studies with Korean university students who exhibited high levels of self-kindness also significantly predicted all aspects of positive mental health and were strongly linked to life satisfaction among community adults [3,27].

The current study offers longitudinal evidence supporting a sequential processing mechanism of self-compassion in relation to mental health outcomes. Specifically, results show that self-kindness indirectly predicts better self-rated mental health through the improvement of common humanity. This finding aligns with theoretical models that view self-compassion as an emotion-regulation strategy, where self-kindness fosters warmth and connectedness, and a sense of common humanity helps buffer against negative self-judgment. The novelty of this study lies in its repeated-measure design within a Hong Kong sample, testing both synergy-based and sequential pathways. Findings extend prior work by showing that self-kindness predicts common humanity, which in turn predicts mental health, thereby refining theoretical models of self-compassion. Notably, this pattern aligns with the coping sequence proposed by [14], in which individuals progress from mindfulness to self-kindness and then to common humanity. While Wong’s model was based on qualitative data, our results extend this framework by providing quantitative support for a key part of this process. Importantly, Hypothesis 2a showed a better model fit and lower AIC compared to Hypothesis 2, reinforcing the idea that prioritizing self-kindness over common humanity in the processing sequence is more valid. This has both practical and theoretical importance, suggesting that interventions aimed at improving mental well-being should build mindfulness to cultivate self-kindness as a foundation for fostering a broader sense of human connection.

Enhancing common humanity may also serve as a downstream effect of self-kindness, reinforcing emotional connectedness and buffering against isolation. This pattern has been noted in prior coping frameworks. This aligns with therapeutic models that emphasize relational meaning-making and alliance-building, particularly in narrative therapy frameworks [28]. In clinical contexts, fostering an emotional connection through compassion may support alliance formation and reduce psychological distress, especially in populations experiencing complicated grief or emotional isolation [29,30].

Although previous research has established that self-compassion contributes to improved mental health, limited knowledge exists regarding the underlying mechanisms linking its negative components—such as self-judgment, over-identification, and isolation—with psychological outcomes. The present study partially supported this relationship: while the overall model was only marginally accepted, all individual pathways were statistically significant, indicating a coherent progression from over-identification to self-judgment and eventually to isolation. This sequential path reflects a maladaptive cycle in which individuals become overly immersed in negative emotional states, engage in harsh self-criticism, and withdraw from perceived social connections. Notably, isolation consistently emerged as a robust negative predictor of self-rated mental health across both time points, highlighting its role as an independent construct rather than simply the absence of common humanity. This finding aligns with theoretical models that frame isolation as a core vulnerability in psychopathology [11]. The persistent sense of disconnection and self-separation, even in the presence of social networks, reflects deeper psychological barriers rooted in self-evaluation processes [12]. Thus, future interventions might gain from specifically tackling internalized self-judgment and perceived isolation as separate therapeutic goals, especially among groups likely to experience emotional over-identification.

These findings also resonate with broader therapeutic literature on grief and emotional processing. For example, [30] propose theoretical and practical guidelines for mental health professionals working with complicated grief, emphasizing the roles of narrative coherence and emotional validation. Their systematic review [29] highlights how narrative therapy can help individuals reconstruct meaning and reduce emotional over-identification, paralleling the maladaptive self-compassion pathways identified in our study. Integrating self-compassion components into such narrative-based approaches may enhance therapeutic outcomes by promoting self-kindness and reducing isolation.

To conclude, over-identification and isolation were found to be associated with poorer self-rated mental health, likely through a cycle of emotional over-engagement, harsh self-judgment, and perceived disconnection. In contrast, self-kindness, particularly when combined with common humanity, was associated with higher mental health ratings over time, supporting a sequential pathway in which kindness fosters a sense of connectedness. The model comparison further suggested that placing self-kindness before common humanity yielded the best explanatory power, shedding light on the directional relationships among the components of self-compassion. Moreover, this study contributes longitudinal evidence to the self-compassion literature and offers culturally relevant insights into how self-compassion functions within a Chinese population.

### Strengths and limitations

The present study employed a repeated-measures design, collecting data at two time points to examine how different components of self-compassion relate to changes in self-rated mental health over time. This design allowed for the application of cross-lagged path analysis, providing insight into the temporal dynamics among self-compassion dimensions. A particular strength lies in the ability to assess directional relationships rather than relying solely on cross-sectional associations. However, the use of convenience sampling may limit the generalizability of the findings. Beyond sampling limitations, methodological constraints should be noted. Path analysis assumes linearity, measurement invariance, and adequate sample size. While these assumptions were tested and met, they nonetheless restrict generalizability. Additionally, reliance on a single‑item measure of mental health may limit construct coverage. In addition, while the analytic model captured key relationships, future research with larger and more diverse samples may further explore complex pathways across multiple time points. We acknowledge that claims of causality are limited. With only two timepoints, temporal ordering can be suggested but not definitively established. Future studies with four or more waves are needed for causal mediation analysis.

### Conclusion and future implications

The current findings highlight the importance of fostering self-kindness and reducing feelings of isolation, both of which were strongly associated with changes in mental health over time. These insights offer valuable guidance for developing interventions. Interventions such as loving‑kindness meditation [31], compassion‑focused therapy [32], and self‑compassionate writing [33] have demonstrated efficacy in cultivating self‑kindness and reducing isolation. These practices may be integrated into community mental health programs to strengthen resilience and well‑being. Practices such as loving-kindness meditation, self-compassionate writing, or compassion-focused therapy may help cultivate self-kindness as a foundation for psychological resilience. Enhancing common humanity may also serve as a downstream effect of self-kindness, reinforcing emotional connectedness and buffering against isolation.

Future studies could evaluate the effectiveness of such interventions using multi-wave longitudinal designs, which would allow for the assessment of sustained change. Additionally, incorporating qualitative interviews may offer deeper insight into participants’ experiences and help tailor intervention strategies to individual needs. Ultimately, these findings may support the integration of self-compassion-based approaches into both clinical practice and community mental health programs, contributing to the prevention and treatment of psychological distress.

## Summary

This study provides longitudinal evidence that self-kindness and common humanity are key pathways to improved mental health, while isolation and over-identification predict poorer outcomes. Although causality cannot be fully inferred, the findings highlight the importance of fostering self-kindness as a foundation for connectedness. Future research should employ multi-wave designs to strengthen causal claims and expand theoretical grounding.
